# Immunological response of human leucocytes after exposure to lipopolysaccharides from *Porphyromonas gingivalis*

**DOI:** 10.1002/cre2.388

**Published:** 2020-12-29

**Authors:** Sara Alizadehgharib, Anna‐Karin Östberg, Agnes Dahlstrand Rudin, Ulf Dahlgren, Karin Christenson

**Affiliations:** ^1^ Department of Oral Microbiology and Immunology University of Gothenburg, The Sahlgrenska Academy, Institute of Odontology Gothenburg Sweden

**Keywords:** *Escherichia coli*, lipopolysaccharide, mononuclear leukocytes, neutrophils, *Porphyromonas gingivalis*

## Abstract

*Porphyromonas gingivalis (P. gingivalis)* is a gram‐negative bacterium and an important etiologic agent of periodontitis. *P. gingivalis* releases outer membrane vesicles containing lipopolysaccharides (LPS), which can penetrate periodontal tissues. Once in the periodontal tissues and in contact with immune cells, it may participate in the destructive innate host response associated with the disease. The exact mechanism of *P. gingivalis* LPS in the disease process is not clear, but it is known to affect a variety of immune responses.

**Objectives:**

To investigate how LPS from *P. gingivalis* affect neutrophil extracellular trap (NET) formation, cell death and production of cytokines from human neutrophils and peripheral mononuclear blood mononuclear cells (PBMCs).

**Materials and methods:**

Isolated neutrophils and PBMCs were cultured with LPS from *P. gingivalis* or *Escherichia coli* (*E. coli*) *(control)*. The NET formation was measured using Sytox green stain. Cell death of neutrophils and PBMCs was analyzed using flow cytometry or Sytox green stain. Cytokine production was measured using enzyme‐linked immunosorbent assay (ELISA) kit or Bio‐Plex assay.

**Results:**

Exposure to LPS from *P. gingivalis* and *E. coli* caused significantly lower cell death in neutrophils. NETs were formed after exposure to the two different LPS. In PBMCs, exposure to *P. gingivalis* and *E. coli* LPS caused increased levels of IL‐1β and IL‐6 compared to unstimulated controls. Increased cell death in PBMCs after exposure to LPS from *E. coli* in comparison to LPS from *P. gingivalis* and unstimulated controls was also observed.

**Conclusions:**

LPS from *P. gingivalis* has the ability to affect both human neutrophils and PBMCs with regard to cytokine production, cell death and production of NETs. LPS from *P. gingivalis* could be involved in the pathogenesis of periodontitis, and our results may contribute information regarding possible markers for diagnosis and targets for treatment of periodontal disease.

Saliva contains 10^7–9^ bacteria/ml (Martin, 2009) consisting of more than 600 different oral bacterial strains (Aas, Paster, Stokes, Olsen, & Dewhirst, 2005; Dewhirst et al., 2010). These bacteria cover all surfaces of the oral cavity including the gingiva and the teeth. The bacteria can be classified in many different ways, for example, based on their cell wall structure as either Gram‐positive or Gram‐negative. In healthy conditions, the oral microbiota maintains a community stability referred to as homeostasis. However, dysbiosis of the microflora caused by changes in the environmental factors may result in different oral diseases such as periodontal disease (Kilian et al., 2016). Periodontal disease is a group of inflammatory diseases that leads to inflammation of the gingiva and destruction of periodontal tissues. Only some bacteria have periodontopathic potential and can initiate periodontal diseases when a critical concentration is reached (Wang & Ohura, 2002). *Porphyromonas gingivalis* (*P. gingivalis*) has been called a “keystone pathogen” associated with periodontitis (How et al., 2016) which leads to disruption of the homeostatic balance with the host tissue, causing destructive inflammation (Hajishengallis & Lambris, 2012).

*Porphyromonas gingivalis* is a proteolytic, gram‐negative bacterium that produces a multitude of different metabolites including various acids, ammonia and hydrogen sulfide (Greabu et al., 2016). These metabolites may induce gingival inflammation which can progress to loss of dental attachment and periodontitis (Hajishengallis, Darveau, & Curtis, 2012). *P. gingivalis* produces other virulence factors such as endotoxins that may cause damage to the host cells. Lipopolysaccharide (LPS) is an endotoxin that is found on the outer membrane of gram‐negative bacteria, and is a potent inducer of immune response by various cell types (Schmalz, Krifka, & Schweikl, 2011). *P. gingivalis* LPS has a critical role in mediating inflammation and inducing cells to secrete pro‐inflammatory cytokines that in turn affect, for example, bone resorption (Diya et al., 2008). Thus, *P. gingivalis* LPS has been considered to be an important pathogenic component in the initiation and development of periodontal disease (Wang & Ohura, 2002).

Gingival crevicular fluid (GCF), flows into the gingival crevice through the junctional epithelium thereby transporting cells into the oral cavity. The population of cells in the GCF comprises mainly neutrophils but also lymphocytes, with fewer T cells than B cells, and monocytes (Attstrom, 1970). It is therefore important to study the effect LPS from *P. gingivalis* may have on target immune cells.

The aim of the present study was to investigate how LPS from *P. gingivalis* affect Interleukin (IL)‐8 production, neutrophil extracellular trap (NET) formation and cell death from human neutrophils. Also, to study the production of IL‐1 receptor antagonist (IL‐1RA), interferon gamma‐induced protein 10 (IP‐10), monocyte chemoattractant protein‐1 (MCP‐1), IL‐1β, IL‐6 and tumor necrosis factor alfa (TNF‐α) and cell death in peripheral mononuclear blood cells. For this purpose, we used LPS from *Escherichia coli* (*E. coli*) as control since it is a potent inducer of immune response by various cell types (Alexander et al., 2001).

## 1 MATERIALS AND METHODS

### 1.1 Isolation of polymorphonuclear neutrophils (PMNs) and peripheral blood mononuclear cells (PBMCs) from human blood

Fresh blood samples from healthy blood donors were obtained from Sahlgrenska University Hospital in Gothenburg, Sweden. Peripheral blood neutrophil isolation was performed as first described by Boyum (Boyum, 1968). After removal of red blood cells in a sedimentation step, the suspension was centrifuged on Ficoll‐Paque Plus (GE Healthcare Bio‐Sciences, Uppsala, Sweden). The pelleted neutrophils and the layer of PBMCs above the Ficoll‐Paque Plus were collected and the remaining erythrocytes were lysed by hypotonic treatment. All the washing of the neutrophils was performed using Krebs Ringer Phosphate (KRG) buffer. In the end, the isolated neutrophils were resuspended in KRG supplemented with Ca^2+^ (1 mM) while the PBMCs were resuspended in phosphate‐buffered saline (PBS), centrifuged, and resuspended in Dulbecco's Modified Eagle's Medium (D‐MEM) (Invitrogen, Lidingö, Sweden) supplemented with 5% heat‐inactivated human AB serum (Sigma Chemical Co., St. Louis, MO), and 100 U/ml of penicillin and 100 μg/ml of streptomycin (Invitrogen, Lidingö, Sweden). Cell viability was determined by staining with 0.4% Trypan Blue (Sigma‐Aldrich) and cells were counted under a microscope using a Bürker chamber.

### 1.2 DNA release measurements from PMNs and cell death measurements in PBMCs using Sytox Green DNA stain

Sytox Green DNA stain (Thermo Fisher Scientific, Gothenburg, Sweden) is a membrane impermeable dye that can be used to measure extracellular DNA from ruptured cells (Gupta, Chan, Zaal, & Kaplan, 2018). For the measurements of NETosis with Sytox Green DNA stain (Thermo Fisher Scientific, Gothenburg, Sweden), cells at a concentration of 0.5 × 10^6^ cells/ml (*n* = 6) in RPMI (without phenol red: Thermo Fisher Scientific) and the Sytox Green DNA stain (2.5 μM; Molecular Probes) were added to a black 96‐well plate. LPS from *P. gingivalis* (InvivoGen, San Diego, CA) and LPS from *E. coli* (serotype O127:B8, Sigma‐Aldrich) at a concentration of 0.1 or 1 μg/ml were added to the wells and the plate was incubated at 37°C and 5% CO_2_. Phorbol 12‐myristate 13‐acetate (PMA; Sigma‐Aldrich) at a concentration of 50 nM/well and 1% Triton X‐100 were used as controls. Sytox Green fluorescence was measured after 0 and 3 hr of incubation at 485/535 nm using a CLARIOstar plate reader.

The same method as mentioned above for measuring NETosis in PMNs, can also be used for measuring cell death in mononuclear cells. For this purpose, Sytox Green DNA stain, mononuclear cells at 0.5 × 10^6^ cells/ml (*n* = 5) together with LPS, 0.1 μg/ml from *P. gingivalis* or *E. coli,* were incubated and measured as mentioned. The cell death was monitored by an increase in fluorescence intensity of the cell‐impermeable dye (Sytox Green) after plasma membrane disintegration.

### 1.3 Microscopic visualization of PMNs

Another method commonly used to confirm the production of NETs is staining the samples with antibodies against myeloperoxidase (MPO). MPO is an antimicrobial protein found on the chromatin fibers released during NETosis.

Isolated human neutrophils (5.5 × 10^5^ cells/ml) were suspended in RPMI and added to poly‐lysine‐coated 24 well glass bottom plates (Cellvis, CA) and incubated at 37°C in 5% CO_2_ for 10 min. After stimulation with PMA (50 nM) or LPS from *P. gingivalis* or *E. coli* (1 μg/ml), the cells were further incubated for 3 hr (the incubation time was chosen from the results received after Sytox Green DNA stain). Cells were fixed in 4% paraformaldehyde for 30 min at room temperature and permeabilized with cold acetone and methanol (1:1) for 5 min. To visualize NETs, the samples were stained with antibodies against MPO (DAKO), followed by secondary antibody staining (Donkey Anti‐Rabbit IgG H&L Alexa Fluor 488 purchased from ThermoFisher). Finally, the coverslips were mounted with ProLong Gold antifade mountant with DAPI (ThermoFisher). The cells were visualized using an Olympus BX41 epifluorescent microscope with the CellSens software.

### 1.4 Evaluation of cell death in PMNs using FACS analyses

For the FACS analyses, 450 μl isolated human neutrophils (5 × 10^6^ cells/ml) (*n* = 6) were suspended in RPMI complemented with 10% FCS and 1% PEST and incubated for 30 min at 37°C in 5% CO_2_. Next, 50 μl of LPS from *P. gingivalis* or *E. coli* (0.1 or 1 μg/ml) (concentrations chosen based on previous studies) were added, the pro‐apoptotic anti‐CD95 (10 μg/ml) were used as positive control.

After 20 hr incubation, 200 μl cell free supernatant from each sample was saved for cytokine analysis, while 200 μl from each cultured sample was washed in 2 ml Annexin buffer [1 mM Hepes, 14 mM NaCl, 0.25 mM CaCl_2_, (pH 7.4)]. The pellets were resuspended in 100 μl Annexin buffer, supplemented with 2 μl Annexin V‐FLUOS and 5 μl 7‐amino‐actinomycin D (7‐AAD) and incubated for 10 min in the dark. Thereafter, another 300 μl Annexin buffer was added, and samples were subjected to FACS analysis using an Accuri C6 (Becton Dickinson, Mountain View, CA). At least 10,000 events were acquired, and neutrophils were gated on the basis of side‐ and forward‐scatter. Apoptosis was assessed on the basis of Annexin V‐FLUOS fluorescence and necrosis was on the basis of membrane permeability to 7‐AAD. All data were analyzed using CFlow and GraphPad Prism software.

### 1.5 Measuring IL‐8 released from PMNs

A DuoSet ELISA Development kit from R&D Systems (Abingdon, UK) was used according to the manufacturer's instructions to measure the levels of IL‐8 in the supernatant fluids of the LPS‐exposed PMN (*n* = 7) cultures.

### 1.6 Bio‐Plex Pro human cytokine assay for cytokine production from PBMCs

The samples (supernatants from PBMCs (*n* = 6) exposed to LPS) were added, and thereafter the color‐coded beads coupled to antibodies. The antibodies reacted with the biomarkers of interest present in the sample. After a series of repeated washes in order to remove un‐bound proteins, a biotinylated detection antibody (used to create a sandwich complex) was added. The final detection complex was formed when streptavidin‐phycoerythrin (SA‐PE) conjugate was added to bind to the biotinylated antibody. Finally, the samples were analyzed using a BioPlex 200 instrument equipped with BioManager analysis software using red and green lasers to detect the different colors on the beads while measuring the fluorescence intensity using a standard curve. The red (635 nm) laser and the green (532 nm) laser measured concentration (pg/ml) and median fluorescence intensity (MFI) respectively. The concentration of the analyte bound to each bead was proportional to the MFI of the reporter signal.

### 1.7 Statistical analysis

All analyses were performed using GraphPad Prism. For all tests, a *p*‐value of <.05 was considered statistically significant. Statistical comparisons between paired samples were made using the Wilcoxon matched‐pairs signed‐rank test.

## 2 RESULTS

### 2.1 Measurement of DNA release from PMNs with Sytox Green DNA stain

**FIGURE 1:**
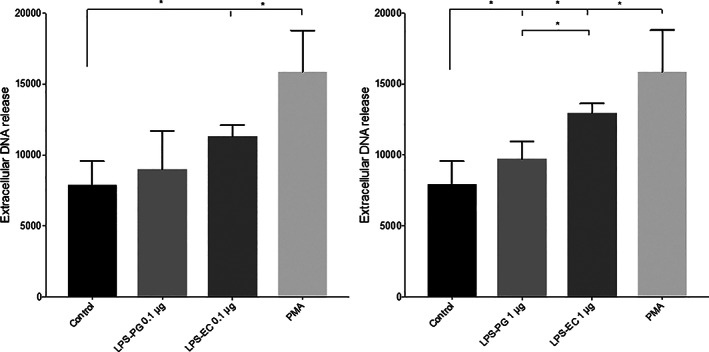
Measurement of DNA release from PMNs after exposure to lipopolysaccharides (LPS) from *Porphyromonas gingivalis* (PG) and *Escherichia coli* (EC) Sytox Green fluorescence was measured from neutrophils cultured with or without LPS (0.1 or 1 μg) from PG or EC for 3 hr. PMA, 50 nM was used as control. Results are from seven independent experiments. *, *p* < .05

As the Sytox Green dye only reacts with extracellular DNA, it has previously, and in combination with microscopy, been used as a proxy for evaluations of NET formation (Makarov et al., 2013). Neutrophils where exposed to LPS (*E. coli* and *P. gingivalis*) for 3 hr. It was observed that 0.1 μg/ml LPS from *E. coli* significantly (*p* = .0312) increased the NETs formation, while exposure to LPS in the same concentration from *P. gingivalis* did not (Figure 1). However, if the LPS concentration was increased to 1 μg/ml, the LPS from both *P. gingivalis* (*p* = .0312) and *E. coli* (*p* = .0156) it significantly increased the amount of NETosis compared to unstimulated cells. LPS from *E. coli* was more potent, indicating towards a higher level of NETosis, as compared to LPS from *P*. *gingivalis* (*p* = .0312) (Figure 1).

### 2.2 Microscopic visualization of PMNs

**FIGURE 2:**
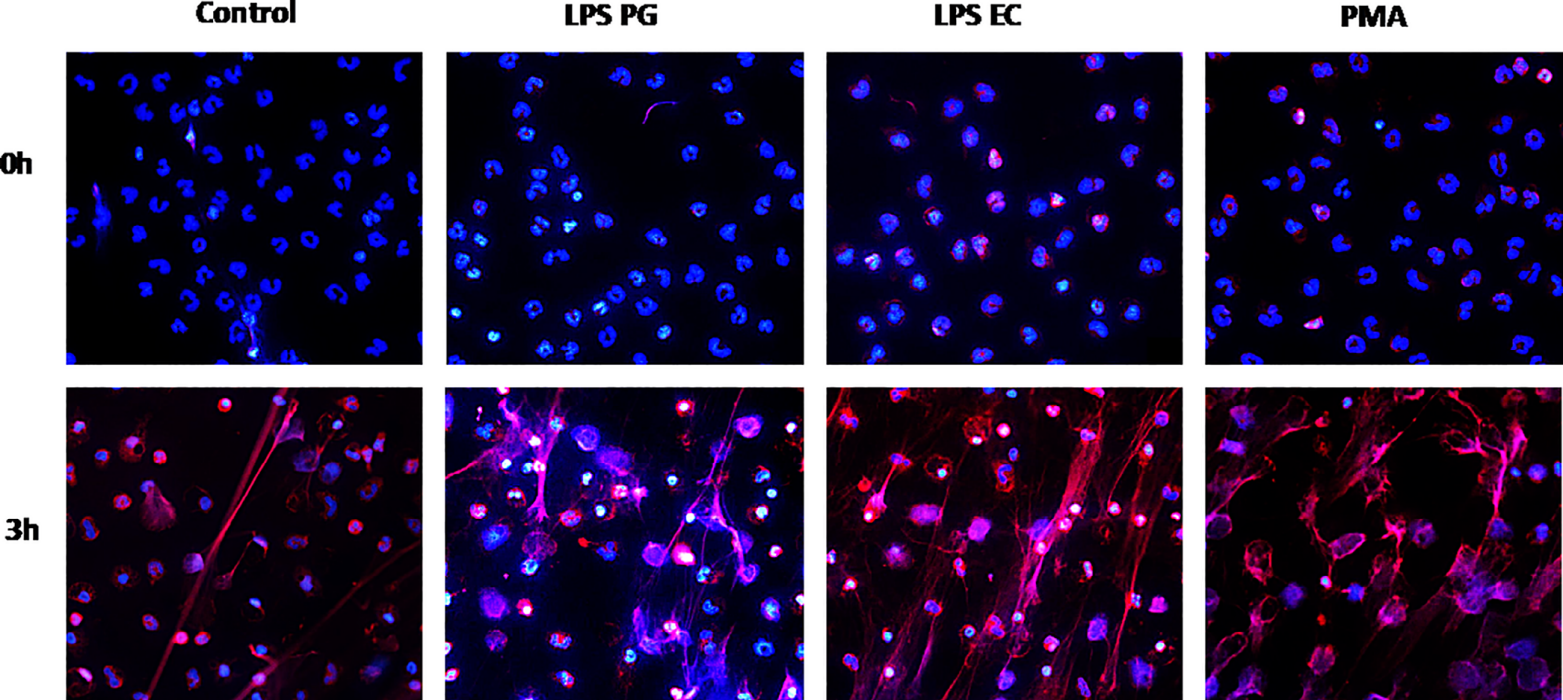
Microscopic visualization of NETs produced by PMNs. Neutrophils were exposed to PMA, 50 nM or LPS from *Porphyromonas gingivalis* (PG) or *Escherichia coli* (EC) for 3 hr incubated at 37°C. The cells were fixed and stained for DNA (blue) and MPO (red). The images are from one representative experiment out of two independent experiments performed. The cells were visualized using an epifluorescence microscope

The results from the Sytox Green plate reader assay was confirmed by using immunofluorescence microscopy and quantifying neutrophils with different nuclear morphology and extracellular DNA after 0 and 3 hr of exposure to LPS (1 μg/ml) (Figure 2).

### 2.3 FACS analyses of cell death in PMNs

**FIGURE 3:**
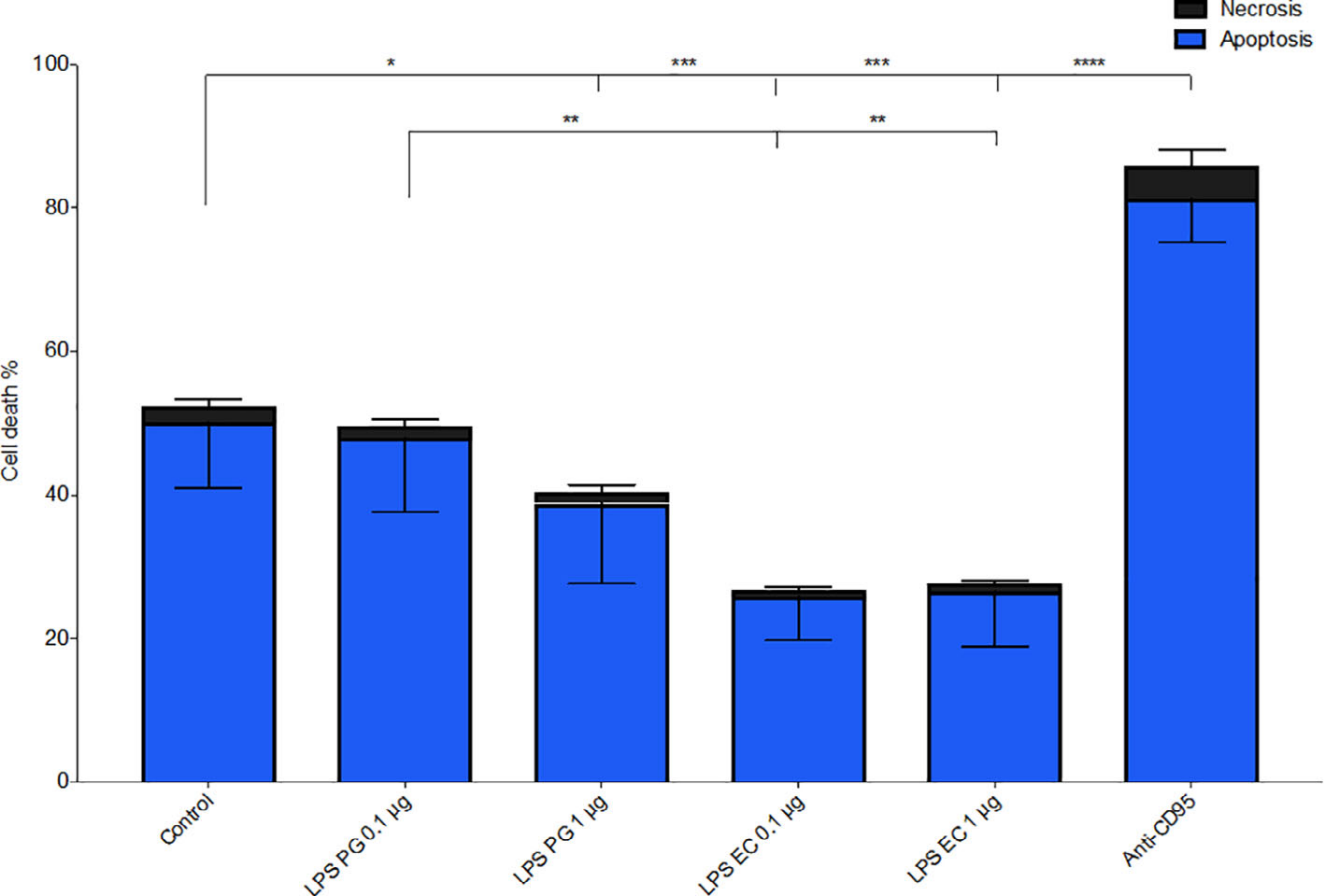
Evaluation of cell death in PMNs using FACS analyses. Neutrophils were exposed to the pro‐apoptotic (anti‐CD95 antibody, 10 μg/ml) and 1 μg/ml LPS from *Porphyromonas gingivalis* (PG) or *Escherichia coli* (EC) for 20 hr and incubated at 37°C. The cell death of the neutrophils was evaluated by flow cytometry. Apoptotic cells labeled with Annexin V are presented as blue parts of the bars, while necrotic cells positive for both Annexin V and 7‐AAD are seen as black parts of the bars. Results are from six independent experiments shown as mean ± *SD*. *, *p* < .05; **, *p* < .01; ***, *p* < .005 and ****, *p* < .001

Out of the cells that were incubated for 20 hr in the absence of stimuli, nearly 50% were apoptotic (Annexin V+/7‐AAD–), while necrosis (Annexin V+/7‐AAD+) was consistently below 5%. The total cell death was significantly lower when cells were exposed to LPS from *E. coli* 0.1 μg/ml (*p* = .0002) and 1 μg/ml (*p* = .0002) compared to unstimulated cells (Figure 3). However, only the highest concentration (1 μg/ml) of LPS from *P. gingivalis* showed anti‐apoptotic effect (Figure 3).

### 2.4 Production of IL‐8 from PMNs

**FIGURE 4:**
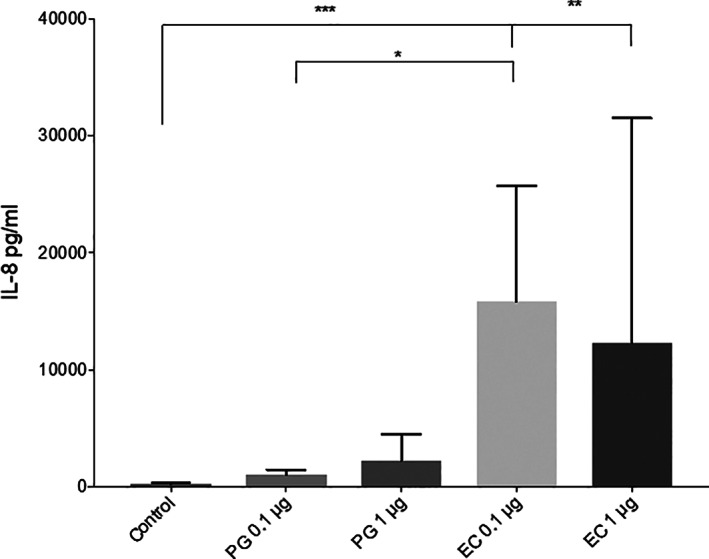
IL‐8 released from PMNs neutrophils were exposed to 0.1 or 1 μg/ml LPS from *Porphyromonas gingivalis* (PG) or *Escherichia coli* (EC) for 20 hr and incubated at 37°C. Total IL‐8 content in the cultures were measured by ELISA. Results are from seven independent experiments. *, *p* < .05; **, *p* < .01; ***, *p* < .005

In order to study IL‐8 production after exposure to the different LPS, supernatants were collected from neutrophils cultured with or without LPS from *P. gingivalis* or *E. coli* for 20 hr. There were significantly higher levels of IL‐8 in the supernatants of cultures exposed to LPS from *E. coli* compared to LPS from *P. gingivalis* 0.1 μg/ml (*p* = .0177), and also between LPS from *E. coli* 0.1 μg/ml (*p* = .0007) and 1 μg/ml (*p* = .0014) and the supernatants of unexposed cells. The release of IL‐8 in response to LPS from *P. gingivalis* did not differ from the unstimulated control (Figure 4).

### 2.5 Cytokine production from PBMCs

**FIGURE 5:**
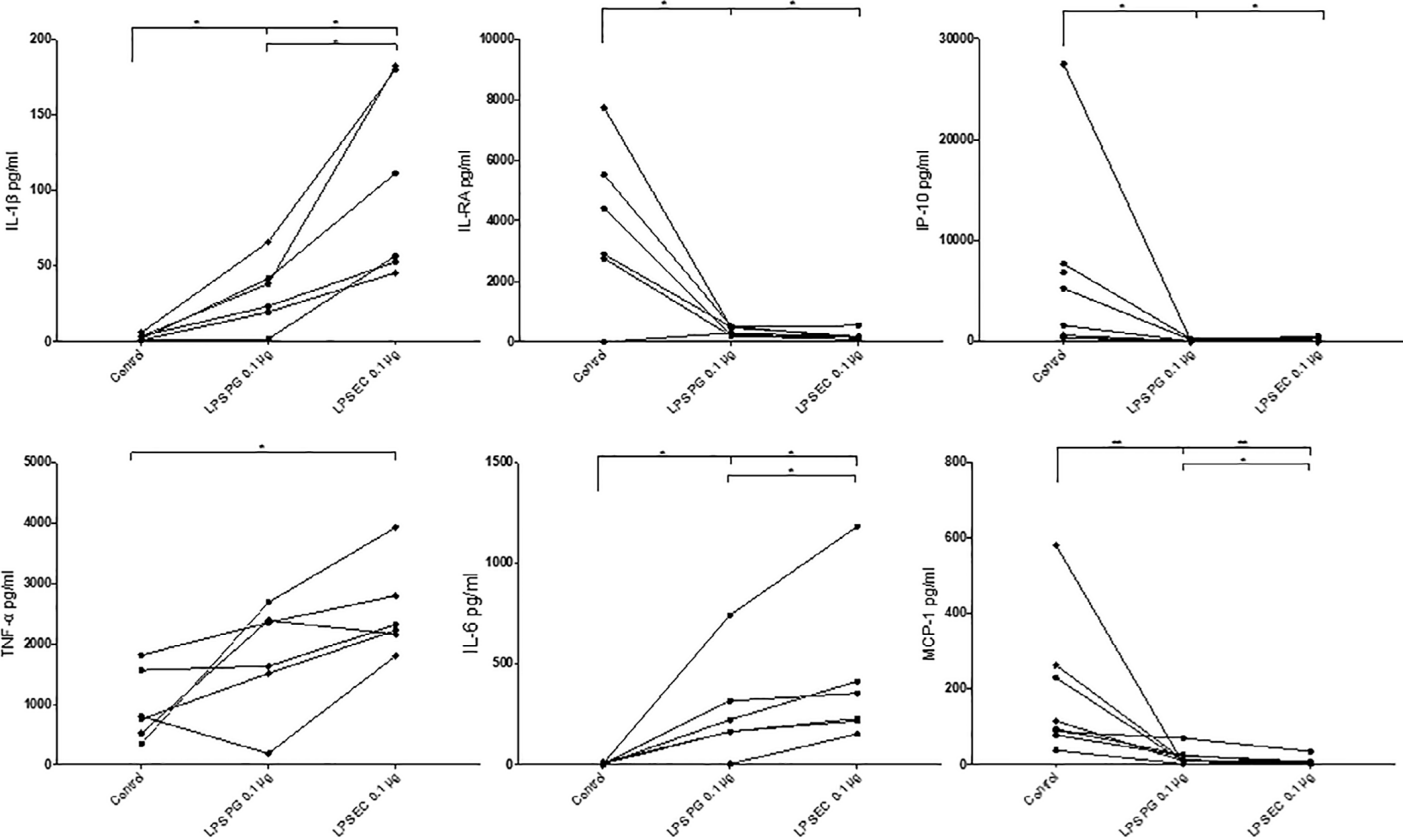
Cytokine production from PBMCs. The levels of six different cytokines were measured in the culture supernatants of PBMCs exposed to 0.1 μg/ml LPS from *Porphyromonas gingivalis* (PG) or *Escherichia coli* (EC) for 20 hr and incubated at 37°C. Results are from six independent experiments. *, *p* < .05; **, *p* < .01

The levels of six different cytokines were measured in the culture supernatants of PBMCs exposed to 0.1 μg LPS from *P. gingivalis* or *E. coli*. After 24 hr of exposure to the two different types of LPS, the levels of the cytokines IL‐1RA, IP‐10 and MCP‐1 were suppressed, while the levels of IL‐1β, IL‐6 and were significantly increased compared to the unstimulated cells (Figure 5). TNF‐α was significantly increased only in the presence of *E coli* LPS. LPS from *E. coli* more potently increased the levels of IL‐1β and IL‐6 in the PBMC cultures compared to LPS from *P. gingivalis* (Figure 5).

### 2.6 Cell death in mononuclear leukocytes using Sytox Green DNA stain

**FIGURE 6:**
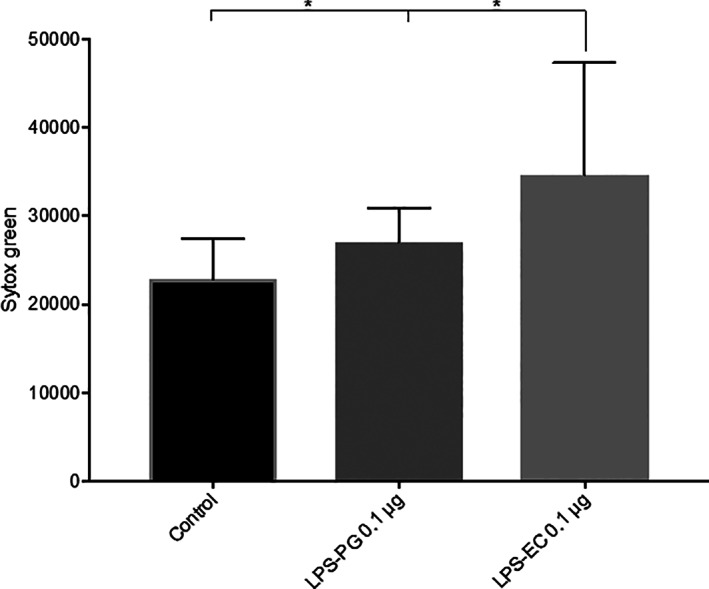
Cell death measurements in PBMCs the PBMCs were exposed to 0.1 μg/ml LPS from *Porphyromonas gingivalis* (PG) or *Escherichia coli* (EC) for 20 hr and incubated at 37°C. Sytox Green DNA stain was used to measure cell death in PBMCs. Results are from six independent experiments. *, *p* < .05

After 24 hr exposure to LPS from *P. gingivalis* and LPS from *E. coli*, PBMC death was measured using Sytox Green DNA stain. The results showed significantly increased cell death in PBMCs after exposure to LPS from *E. coli* in comparison to the LPS from *P. gingivalis* (*p* = .0312) and to unstimulated cells (*p* = .0312) (Figure 6).

## 3 DISCUSSION

*Porphyromonas gingivalis* is a Gram‐negative bacterium recognized as an important etiological agent of chronic periodontitis and can be found in a large proportion of the human population (Holt, Kesavalu, Walker, & Genco, 1999). A major constituent of the outer membrane of *P. gingivalis* is LPS which plays a critical role in mediating inflammation and inducing various immune cells to secrete pro‐inflammatory cytokines (Yucel‐Lindberg et al., 2013), including IL‐1β, TNF‐α and IL‐6 (Diya et al., 2008). It has been demonstrated that most LPS stimulate the production of pro‐inflammatory cytokines mainly through Toll‐like receptor 4 and nuclear factor‐κB (Diya et al., 2008). However, *P. gingivalis* LPS has been shown to differ from *E. coli* in structure and various functional activities (Diya et al., 2008; Holt et al., 1999). In PMNs stimulated with *E. coli* LPS there has been a greater production of IL‐1β, IL‐1RA, IL‐8 and TNF‐α compared to exposure to *P. gingivalis* LPS (Yoshimura, Hara, Kaneko, & Kato, 1997). However, in most publications LPS from *E. coli* is used to investigate immunological responses in various cell types and inflammatory conditions, but in oral infections LPS from oral microbes as *P. gingivalis* is of more interest. Some of the responses that have been studied in the present study are cell death processes in PMNs and PBMCs, the effect on NETosis, the release of IL‐8 from PMNs and the production of pro‐inflammatory cytokines produced by PBMCs.

Periodontal diseases are multifactorial, perhaps beginning with the activation of the immune system at the cellular level by the LPS from a potential pathogen such as *P. gingivalis*. Neutrophils are the most abundant cells in the gingival pockets in periodontal disease (Scott et al., 2012), but it is obvious that there are also multiple other cells involved in the immune responses caused by LPS. Therefore, it was of interest for us to investigate the effects caused by LPS from *P. gingivalis* on neutrophils and other leukocytes that are responsible for the immune system activation by *P. gingivalis* LPS. Neutrophils are primary effectors of the innate immune system against microbial pathogens. In addition to phagocytic killing, neutrophils can catch and kill microbes via an alternative mechanism known as NET formation also called NETosis. NETosis is known to be a violent, pro‐inflammatory type of death where NETs are being thrown out from the cells in order to capture the pathogen (Remijsen et al., 2011). NETs are networks composed of chromatin and neutrophil granule proteins with high bactericidal potential. NETs are thought to neutralize pathogens and create a barrier that prevents the spread of bacteria (Brinkmann et al., 2004). It has previously been shown that LPS from *E. coli* has the potential to induce NETosis (Brinkmann et al., 2004). There have been several studies investigating the role of NETosis in periodontitis (Vitkov, Hartl, Minnich, & Hannig, 2017; White, Chicca, Cooper, Milward, & Chapple, 2016). The proteolytic enzymes, called gingipains from *P. gingivalis* have been shown to have a huge role in the NETosis triggered by this bacterium (Bryzek et al., 2019). However, no previous studies have been conducted on LPS from *P. gingivalis* and its effect on NET formation. In the present study, it has been shown that neutrophil exposure to LPS from *P. gingivalis* significantly increased the release of extracellular DNA and the formation of NETs was confirmed microscopically. However, LPS from *E. coli* was observed to be a more potent NET inducer as compared to LPS from *P. gingivalis*.

Another observed effect of by LPS exposure on the neutrophils was the increased IL‐8 production. IL‐8 is a chemokine with proinflammatory abilities inducing neutrophil recruitment (Baggiolini, Loetscher, & Moser, 1995). This recruitment might result in a prolonged inflammation and may play a role in periodontal disease that is a chronic inflammatory condition. As previously shown, neutrophils exposed to LPS from *E. coli* release high levels of IL‐8 (Christenson et al., 2013; Yoshimura et al., 1997) while the results of our study show that exposure to LPS from *P. gingivalis* only tended to slightly increase the level of IL‐8, however none of statistically significance value.

Previous studies conducted on monocytes/macrophages have shown that exposure to LPS from *P. gingivalis* induce production of IL‐1β, TNF‐α and IL‐6 (Baqui et al., 1998; Diya et al., 2008). These cytokines are usually increased once monocytes or macrophages are stimulated with LPS. However, it has been reported in most studies that *P. gingivalis* LPS is less potent than *E. coli* LPS in inducing the release of inflammatory cytokines in various cells (Diya et al., 2008; Martin, Katz, Vogel, & Michalek, 2001). This is in concordance with the results in the present study. It was also observed that after 24 hr exposure of the two different LPS, the levels of the cytokines IL‐1RA, IP‐10 and MCP‐1 were suppressed, while the levels of IL‐1β, IL‐6 and TNF‐α were significantly increased. There were also significantly higher levels of IL‐1β, IL‐6 and a tendency towards higher level of TNF‐α in the PBMC cultures exposed to LPS from *E. coli* compared to LPS from *P. gingivalis*. TNF‐α is a proinflammatory cytokine that is involved in upregulation of inflammatory reactions. This may explain why LPS from *E. coli* is known to be more potent than LPS from *P. gingivalis*. Exploring the effects of the two LPS on human PBMCs, the viability of these cells was measured using Sytox Green DNA stain. After 24 hr exposure, the results showed significantly increased fluorescence intensity in cells exposed to LPS from *E. coli* in comparison to the LPS from *P. gingivalis* and cells alone. In summary, after 24 hr exposure, LPS from *E. coli* caused higher cell death in human PBMCs compared to LPS from *P. gingivalis*.

The immunomodulating properties of LPS from *P. gingivalis* were not as strong as the those of LPS from *E. coli*. The ability of *P. gingivalis* LPS to affect the different parts of the immune system may explain its possible participance in the pathogenesis of periodontitis. While the viability of mononuclear cells was unaffected by exposure to LPS from *P. gingivalis*, the proinflammatory cytokine production (IL‐1β, IL‐6 and TNF‐α) was elevated and the anti‐inflammatory cytokine IL‐1RA was decreased. The change in the balance of the cytokines might promote the inflammation caused by LPS from *P. gingivalis*. This can be related to the previously reported ability of *P. gingivalis* to cause inflammation of the gingiva (gingivitis) that might result in chronic destruction of connective tissues and thereby the formation of periodontal pockets and ultimately result in loss of teeth (Hajishengallis et al., 2012). Another interesting finding in this study was the ability of LPS from *P. gingivalis* to cause NETosis in human PMNs. As summarized by Rajendran et al., the literature has recently shown that NET production has a significant role in periodontal infections (Rajendran et al., 2018). The changes in the normal functions of neutrophils and PBMCs caused by LPS from *P. gingivalis* exposure may lead to an altered inflammatory response.

In the present study we have put focus at describing some of the effects caused by LPS *P. gingivalis* exposure, and a limitation of the study is that we cannot describe the exact mechanism behind these effects. However, in order to be able to describe an exact mechanism, the present study was required as a first step.

In conclusion, LPS from *P. gingivalis* is an immunomodulator that has the ability to affect both human neutrophils and PBMCs. These effects included modulated cytokine production, affected cell death and production of NETs. Although the results showed a clear change in immunological response due to LPS from *P. gingivalis,* the changes were less compared to those caused by LPS from *E. coli*. The present findings may contribute as future targets for treatment of immune reactions caused by LPS from *P. gingivalis*.

## CONFLICT OF INTEREST

The authors declare that they have no conflict of interest.

## ETHICS STATEMENT

The peripheral blood cells obtained from Sahlgrenska University Hospital blood bank are de‐identified, and according to the Swedish legislation section code 4§ 3 p SFS2003:460, no informed consent is needed.

## Data Availability

Data available on request from the authors
